# The Disinfection of Excreta

**Published:** 1898-04-23

**Authors:** 


### THE DISINFECTION OF EXCRETA.

It is not always safe to draw deductions from laboratory experiments in regard to the efficiency of disinfectants under ordinary conditions. Especially is it unsafe to take the action of various agents upon micro-organisms contained in clear broth cultures as a guide to their effect upon the mixed and varied substances contained in the excreta. Mr. C. A. Hill and Dr. J. H. Abram, of Liverpool, have then done well in performing their experiments under exactly the same conditions as are met with in practice. The first thing they insist on is that the fæces must be thoroughly mixed with the substance employed, the most powerful disinfectants being entirely without effect unless complete mixture is ensured. Then the mixture should stand for at least half an hour, the experiments all being made on that basis. If these conditions are observed, carbolic acid, crude carbolic acid, formol, creolin, chinosol, and corrosive sublimate are all effective. The following are the minimum strengths which should be used: Carbolic acid, 1 in 20; crude carbolic, 1 in 40; formol, 1 in 40; chinosol, 1 in 600; creolin, 1 in 40; mercuric chloride, 1 in 500. Any of these six disinfectants may, they say, "be regarded as absolutely certain in their results when thoroughly mixed with the stool and allowed to stand for half an hour." There are, however, certain objections to some of them. Mercuric chloride is a scheduled poison, and it acts upon the metal work of the drainage system; moreover, by producing a red colouration with stercobilin, it may mask the presence of blood in a typhoid stool. It also coagulates albumen, and may thus protect organisms enclosed in the centre of the coagulum. Crude carbolic is cheap and efficient, but it does not mix well with the fæces, and it stains linen and is poisonous. Formol and creolin are good, but are rather costly. The remaining substance, chinosol, appears to be the best. It is reliable, is an excellent deodorant, and mixes well with the fæces. Although it is dearer than crude carbolic, its cost is counterbalanced by its portability, as it is put on the market in tablet form, so that an effective solution can be easily obtained wherever it may be required.

